# Selective Caries Removal in Permanent Teeth (SCRiPT) for the treatment of deep carious lesions: a randomised controlled clinical trial in primary care

**DOI:** 10.1186/s12903-021-01637-6

**Published:** 2021-07-09

**Authors:** Jan E. Clarkson, Craig R. Ramsay, David Ricketts, Avijit Banerjee, Chris Deery, Thomas Lamont, Dwayne Boyers, Zoe Marshman, Beatriz Goulao, Katie Banister, David Conway, Bhupinder Dawett, Sarah Baker, Andrea Sherriff, Linda Young, Marjon van der Pol, Graeme MacLennan, Ruth Floate, Hazel Braid, Patrick Fee, Mark Forrest, Jill Gouick, Fiona Mitchell, Ekta Gupta, Riz Dakri, Jennifer Kettle, Tina McGuff, Katharine Dunn

**Affiliations:** 1 Dental Health Services Research Unit, Dundee Dental School, The University of Dundee, 9th Floor, Park Place, Dundee, DD1 4HN UK; 2 NHS Education for Scotland, Edinburgh, UK; 3 Health Services Research Unit, University of Aberdeen, Aberdeen, UK; 4 Faculty of Dentistry, Oral and Craniofacial Services, Kings College London, London, UK; 5 School of Clinical Dentistry, University of Sheffield, Sheffield, UK; 6 Health Economics Research Unit, University of Aberdeen, Aberdeen, UK; 7 School of Medicine, Dentistry and Nursing, University of Glasgow, Glasgow, UK; 8 Hafren House Dental Practice, Alfreton, Derbyshire UK

**Keywords:** Selective caries removal, Complete caries removal, Primary care, Randomised controlled trial, Partial caries removal, Minimally invasive dentistry, Patient-centred outcomes, Oral-health-related quality of life, Willingness to pay, Cost–benefit analysis

## Abstract

**Background:**

Dental caries is one of the most prevalent non-communicable disease globally and can have serious health sequelae impacting negatively on quality of life. In the UK most adults experience dental caries during their lifetime and the 2009 Adult Dental Health Survey reported that 85% of adults have at least one dental restoration. Conservative removal of tooth tissue for both primary and secondary caries reduces the risk of failure due to tooth-restoration, complex fracture as well as remaining tooth surfaces being less vulnerable to further caries. However, despite its prevalence there is no consensus on how much caries to remove prior to placing a restoration to achieve optimal outcomes. Evidence for selective compared to complete or near-complete caries removal suggests there may be benefits for selective removal in sustaining tooth vitality, therefore avoiding abscess formation and pain, so eliminating the need for more complex and costly treatment or eventual tooth loss. However, the evidence is of low scientific quality and mainly gleaned from studies in primary teeth.

**Method:**

This is a pragmatic, multi-centre, two-arm patient randomised controlled clinical trial including an internal pilot set in primary dental care in Scotland and England. Dental health professionals will recruit 623 participants over 12-years of age with deep carious lesions in their permanent posterior teeth. Participants will have a single tooth randomised to either the selective caries removal or complete caries removal treatment arm. Baseline measures and outcome data (during the 3-year follow-up period) will be assessed through clinical examination, patient questionnaires and NHS databases. A mixed-method process evaluation will complement the clinical and economic outcome evaluation and examine implementation, mechanisms of impact and context. The primary outcome at three years is sustained tooth vitality. The primary economic outcome is net benefit modelled over a lifetime horizon. Clinical secondary outcomes include pulp exposure, progession of caries, restoration failure; as well as patient-centred and economic outcomes.

**Discussion:**

SCRiPT will provide evidence for the most clinically effective and cost-beneficial approach to managing deep carious lesions in permanent posterior teeth in primary care. This will support general dental practitioners, patients and policy makers in decision making.

*Trial Registration *Trial registry: ISRCTN. Trial registration number: ISRCTN76503940. Date of Registration: 30.10.2019. URL of trial registry record: https://www.isrctn.com/ISRCTN76503940?q=ISRCTN76503940%20&filters=&sort=&offset=1&totalResults=1&page=1&pageSize=10&searchType=basic-search.

**Supplementary Information:**

The online version contains supplementary material available at 10.1186/s12903-021-01637-6.

## Background

Dental caries in permanent teeth is a widespread and costly public health problem. Globally, it is one of the most prevalent non-communicable disease and can have serious health sequelae which can impact negatively on quality of life and productivity[1–5]. In the UK most adults experience dental caries during their lifetime and the 2009 Adult Dental Health Survey reported that 85% of adults have at least one restoration [6]. The removal of less tooth tissue for both primary and secondary caries reduces the risk of failure due to tooth-restoration fracture, complex fracture and the surfaces are less vulnerable to further caries [7]. Furthermore, smaller cavities simplify the operative procedure when placing restorations, so improving their long-term viability.

However, despite the prevalence of this non- communicable disease there is no consensus about how much caries affected tooth tissue to remove prior to placing a filling to achieve optimal patient outcomes. Evidence for selective compared to complete or near-complete caries removal suggests there may be benefits for selective removal in sustaining tooth vitality therefore avoiding abscess formation and pain, and eliminating the need for more complex, costly treatment or eventual tooth loss. However, the evidence is of low scientific quality and mainly gleaned from studies on primary teeth.

The majority of current NHS treatment is managing failed restorations and their consequences moving to increasingly invasive and costly treatments. NHS expenditure on primary and secondary dental care in England approximates to £3.4bn per year, with over one million patient contacts weekly. Many of these contacts relate to treatment of caries. In 2016/17 in England, there were over 7m permanent fillings, over 2m extractions and around 1.5 m endodontic treatments, crowns, inlays or bridges provided to adult NHS patients in primary dental care. The total value of these treatments was about £1.27 bn, with a significant burden on both patients (~£635 m) and the NHS (~£639–£290 m for fee-paying and a further £349 m for exempt patients) [8] . A similar pattern of spend has been observed in Scotland for 2017 [9]. This does not take into account the dental treatment provided in private practice or the costs of NHS treatments delivered in secondary care.

The importance of this topic has been highlighted both by patients and general dental practitioners. General dental practitioners within the Scottish Dental Practice Based Research Network (http://www.sdpbrn.org.uk/) in 2011 voted this as the top research priority. More recently, focus groups and interviews with general dental practitioners followed by a national online survey of dental practitioners, which was conducted as part of this proposal, have established the current importance of the topic and informed the trial design of SCRIPT. The 320 survey participants demonstrated considerable variation in practice with 3% reporting they always perform selective caries removal and 13% reporting they never perform selective carious tissue removal. The primary outcome in the SCRIPT trial was considered to be important by 99% of participants and their free text comments clearly demonstrate the current professional uncertainty around how best to manage patients with deep carious lesions—“*would be willing to use selective caries removal if there was proof it does no harm” and “I'd be very keen to see more research in this area and would put it to use in my day to day practice.”*

In this trial, in addition to adults, young people between the ages of 12 years and 15 years will be included because the 2013 UK child dental health survey reported that nearly a half (46%) of 15 year olds and a third (34%) of 12-year-olds had "obvious decay experience" in their permanent teeth^12^. Of these a fifth (19%) of 12-year-olds and 15-year-olds (21%) had decay into dentine requiring treatment. Toothache was experienced by 18% of 12-year-olds and 15% of 15-year-olds. This suggests that the burden of dental caries is very high in this group and therefore including this age group in the trial would generate evidence of acceptability and potential issues related to actual treatment provision. Such evidence would help the dental practitioners in primary care to adopt minimally invasive approaches to carious tissue removal especially when the recent EU directive has recommended not to use amalgam fillings in under 15-year-olds [10].

This was further highlighted by a research priority setting exercise conducted with patients at a general dental practice in Derbyshire with the greatest potential benefit seen for the young people. A Patient and Public Involvement (PPI) group at the Health Services Research Unit (HSRU) in Aberdeen felt that development of such conservative techniques was very important as they could improve both the health and dental experience of patients. Overall, new evidence investigating whether a procedure involving sealing in decay in reduces the chances of teeth being lost and reduces the chance of painful episodes and further complex treatment like root canal treatment would be important and welcomed by patients.

Randomised controlled clinical trials and systematic reviews have concluded that selective caries removal (SCR) may offer benefits, but the evidence on managing deep caries in permanent teeth is weak and biased [11–14]. Schwendicke et al. [11] found a significant risk reduction for pulp exposure (OR 0.31 [0.19–0.49]), but pulp symptoms (OR 0.58 [0.31–1.10]) and risk of restoration failure (OR 0.97 [0.64–1.46]) were inconclusive (n=1,257). Similarly, for selective caries removal, Ricketts et al. [14] found a significant risk reduction for pulp exposure (OR 0.23 [0.08–0.69]), but again pulp symptoms (OR 0.27 [0.05–1.60]) and risk of failure were inconclusive (n=345 in the partial caries analysis of primary and permanent teeth). Quality of the evidence is judged to be poor scientifically and most of the included studies were conducted solely on children with short term follow-up [15–17], used less durable materials [16, 18] or included more aggressive complete caries removal (CCR) interventions to “hard” dentine [13, 19] than would be present in UK NHS.

Bjørndal et al. [12] followed-up for 5 years a 2-staged (stepwise) carious tissue removal protocol on lesions spreading into the pulpal quarter of dentine (n=239). Pulp exposure rate was lower in this stepwise removal protocol (21.2% vs. 35.5%; p = 0.014) and more successful (60.2% vs 46.3%) (p = 0.031) when pulp exposures were classed as failure. The trial has limited generalizability to SCRIPT’s context and patients: it recruited participants with more severe caries and its two interventions differed from SCRIPT’s. Their partial approach (2-step) was similar to CCR; their CCR approach was more aggressive than the one we propose to use, excavating “hard” dentine. Therefore, more evidence is needed about the performance of a minimally invasive option of SCR in the NHS and about whether dentists will adopt it. There is one ongoing trial in France [20] looking at this research question in a secondary care setting. Personal communication with the trial team confirmed they had experienced a merger (contamination) of the two technologies (i.e. the two techniques undertaken by an individual dentist tended to become more similar dependent upon their initial skills and preferences). The trial has had more than 30% loss to follow-up. The authors believe that is due to the secondary care setting where patients are usually transient and short term. Our research robustly monitors and minimises the risk of the technologies merging, and most patient participants are routine attenders at their dental practice.

Therefore, this research is important to establish whether SCR will sustain tooth vitality and reduce the need for complex, costly treatment including root canal treatment, crowns, extractions, bridgework or implants [21]. In addition to potential cost savings, this reduced treatment need would be expected to improve quality of life and reduce anxiety and stress.

The risk to participants is low as both treatments are utilised in NHS practice albeit that SCR is relatively under-employed. For SCR, which is a minimally invasive treatment, the perceived risk is the residual caries alone may lead to further deterioration of the tooth. The risks from CCR are already managed within existing dental practice so the trial does not impose any new risks. Risk in both arms will be monitored as part of the trial as the primary and some secondary outcomes directly measure the impact of each treatment.

## Methods/design

This is a pragmatic, primary dental care, multi-centre, single-masked, two-arm patient randomised controlld trial including an internal pilot, comparing the clinical-effectiveness and cost–benefit of Selective Caries Removal (SCR) with Complete Caries Removal (CCR) in permanent posterior teeth in participants aged 12 years and over.

**Fig. 1:**
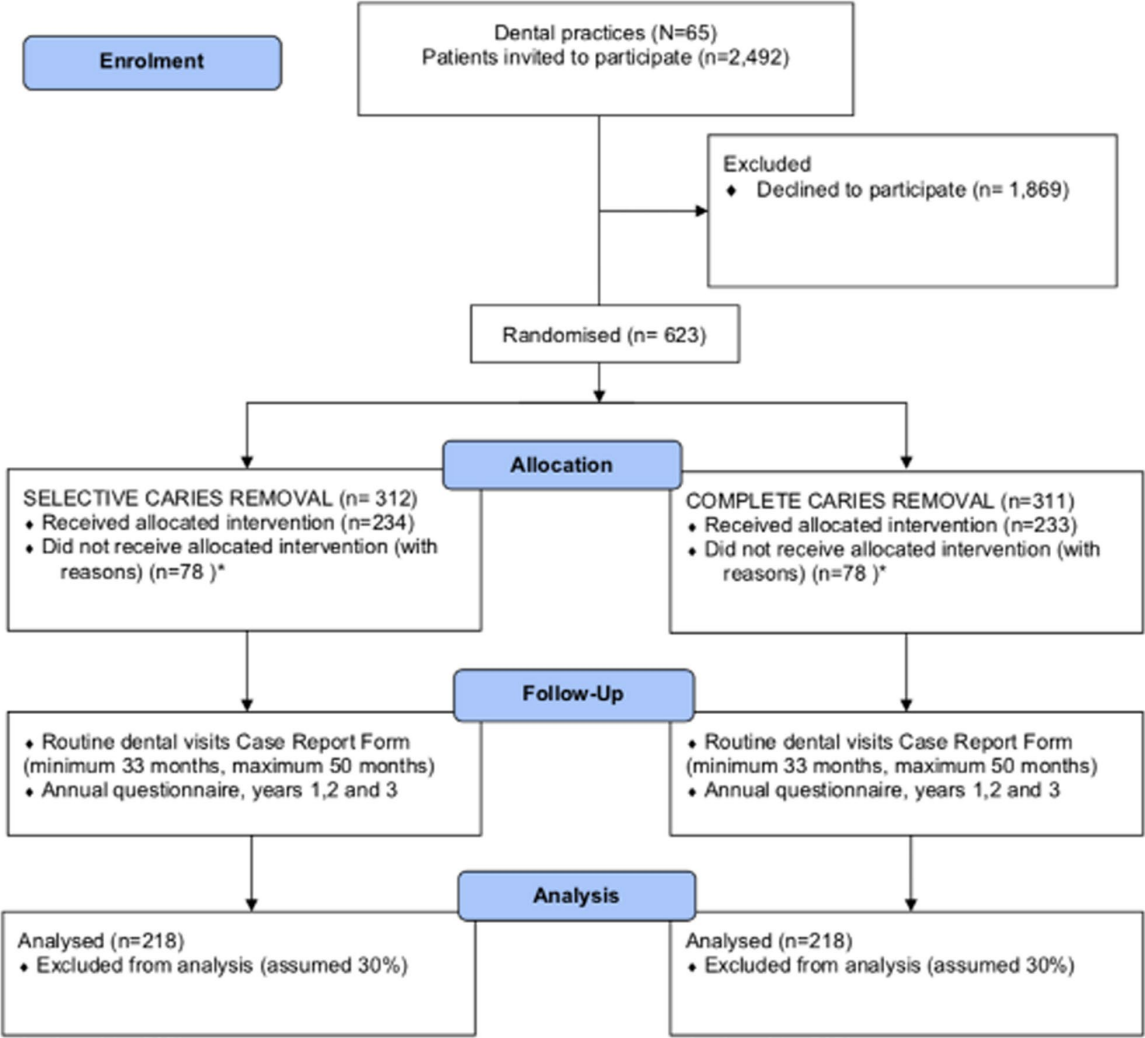
A flow diagram illustrating the SCRiPT Trial design. *The study is statistically powered so that up to 25% of participants in total could receive the non-allocated procedure. Processes are in place to minimise this from occurring

Follow-up will last at least 3 years and will be conducted in multiple sites across the UK. The design includes an internal pilot to assess the recruitment of practices and participants and monitor compliance with the clinical protocol and acceptability for patients and clinicians (Fig. 1).

### Objectives

#### Primary objective

To compare clinical effectiveness and cost-benefit (modelled over a lifetime horizon) of selective caries removal (SCR) with complete caries removal (CCR) in permanent teeth in NHS dental attenders aged 12 years and over who have deep caries in an adult pre-molar or molar tooth.

#### Secondary objectives

- To evaluate pulp exposure during caries removal, progression of caries, dental pain and need for dental pain relief, restoration failure and patient oral health-related quality of life.
- To determine general population preferences for the type of treatment provided and outcomes of care and predict general population barriers to its uptake. To estimate NHS and patient perspective costs, incremental net benefit (willingness to pay minus costs) over three year follow up and incremental cost per Quality Adjusted Life Year (QALY) gained over the three year follow-up and a modelled lifetime horizon.
- To explore the implementation of technologies and the mechanisms of impact including acceptability

### Interventions

The interventions being evaluated, SCR and CCR, differ solely in the amount of carious dentine removed during the excavation phase of restorative treatment of deep dental caries. Clinicians will restore the tooth with the material that they would normally use. This may be amalgam or resin composite with or without glass ionomer cement. Placement of a separate pulp lining protection material is permitted. However, it should not be medicated i.e. including steroids or antibiotics. Information about any pulp protection material placed will be recorded.

#### Intervention: selective caries removal (SCR)

- Gain access to the dentine caries by removing superficial enamel or existing restoration
- Remove caries from the periphery of the cavity to allow for good adaptation and seal to the restoration
- Either at the enamel dentine junction or the peripheral 2 mm of dentine if the cavity margin is on root dentine. Remove remaining carious dentine to soft dentine “that deforms when an instrument is pressed into it and can be easily scooped up (e.g. with a spoon hand excavator) with little force being required” [22]

#### Control: complete caries removal (CCR)

- Gain access to the dentine caries by removing enamel or existing restoration
- Remove caries to firm dentine “physically resistant to hand excavation and some pressure needs to be exerted through an instrument to lift it” [22]

#### Training in the delivery of intervention

As the primary difference between the health technologies being assessed is the amount of carious dentine removed effective clinical training is of prime importance. The training will utilise a novel clinical learning tool, providing an on demand online resource and mechanisms for monitoring sustained delivery of the allocated technology.

All clinicians will participate in and complete the SCRiPT trial training (Part 1 hands-on in clinical procedures Part 2 Good Clinical Practice). Other members of the dental team will complete SCRIPT Part 2 which can be delivered remotely. Prior to the COVID-19 pandemic Part 1 training was provided in its entirety at hands-on clinical facilities. This included information on the background of operative caries management and trial interventions, together with hands on practice of both SCR and CCR on plastic teeth that have been 3D printed at different densities to replicate caries.

During the COVID-19 pandemic Part 1 training was re-designed to meet physical distancing guidelines by using a blended learning approach i.e. a combination of virtual theoretical training and a remote practical training exercise. Part 1 training was divided into part 1a and 1b. Part 1a covers the background to the interventions and description of the interventions, including video recordings of the interventions. This training was/is delivered remotely via Zoom meetings. Part 2a involves posting 3D plastic teeth to the dentists to complete the trial interventions and returning a selected pair of teeth to the trial clinical team for validation and to check adherence to protocol. In cases where further training was required this was followed up by individual zoom discussion, feedback and further practice on 3D printed teeth.

### Monitoring fidelity to protocol

Monitoring fidelity with the clinical protocol is essential to exclude merger of the clinical techniques and consistency of technology used when more than one tooth has a deep carious lesion. We will implement 3 strategies to assess compliance:

- Clinicians will be asked after every third patient to cut both a SCR and CCR cavity in two 3D computer generated sample teeth and return these to the research office. These teeth will be examined by a member of the clinical team to monitor compliance. If merger of SCR and CCR is observed additional training and or reinforcement of protocol will be provided
- The CRF will include an assessment of colour, hardness and consistency to monitor whether SCR or CCR has been performed according to protocol. The lighter shade would be consistent with CCR, a darker shade with SCR. The hardness of the cavity will be assessed with a dental explorer/spoon excavator. Soft would indicate SCR and hard would indicate CCR. The CRFs will be assessed in real time to monitor fidelity and feedback will be provided if evidence of merger is observed.
- Baseline and 1-year follow-up bitewing radiographs will be assessed by a researcher masked to treatment group, to confirm the extent of initial caries and following the restoration the presence of caries. Follow up x-rays will be taken at intervals judged appropriate by the clinicians therefore assessment will be 6–12 months after treatment.

### Study recruitment and allocation

#### Recruitment of dentists

General dental practice is the main provider of NHS dental care. SCRIPT will recruit approximately 65 NHS dental practices (and up to 75 clinicians) from across the UK within the participating regions. A list of study sites will be available on the study website.

An open invitation will be distributed using routine NHS communication systems such as NHS Education for Scotland’s Portal, BDA section and Local Dental Committee networks to request expressions of interest. Practices that have successfully recruited to and delivered previous dental HTA trials will be targeted through our research networks. Also, the planned NIHR Clinical Research Network oral and dental specialty national questionnaire will be used to identify interested practices. Following the expression of interest, a trial briefing session will be delivered remotely, and an appraisal of each practice’s ability to recruit participants will be conducted.

#### Identifying and recruiting participants

The trial will aim to recruit 65 practices (each recruiting an average of 10 participants) to achieve the target of 623 participants. Trial clinicians will identify patients at routine visits that meet the inclusion criteria and explain the trial. Eligible patients who express interest will be given a participant information leaflet (Additional file 1) and an appointment for their treatment as per current clinical practice. At the treatment visit the patient will be given the opportunity to clarify any questions prior to informed consent being obtained and recorded on a written consent form (Additional file 2).

The eligibility of all participants will be assessed and determined by their dental practitioner following clinical and radiographic examination according to pre-determined criteria. Those who do not wish to take part will receive caries removal and restoration as per current practice. Those who are eligible and wish to take part will give informed consent to the clinician providing treatment.

A baseline questionnaire will be completed by the participant to collect baseline patient centered outcomes. Randomisation will then take place using an online randomisation system. If the participant has more than one eligible tooth, a trial tooth will be selected at random by the randomisation application. The randomisation algorithm will use recruitment site, number of eligible teeth (1; > 1) and type of caries (primary and secondary) as minimisation covariates to allocate to treatment intervention and control groups in a ratio of 1:1To maintain participant blinding, participants will not be informed of their allocated treatment group following randomisation. Baseline clinical information will be recorded by the clinician after the treatment has been provided.

After the initial intervention participants will receive any treatment deemed clinically appropriate by their dentist as per normal practice.

#### Inclusion criteria

- Aged 12 years and over, suitable to receive either clinical procedure.
- Patients able to provide informed consent
- Patients receive some or all of their treatment under NHS
- One (or more) pre-molar or molar teeth with caries (primary or secondary) extending into the pulpal third of dentine.
- Caries may be proximal and/or occlusal and the lesion will be suitable for a direct filling with a single restoration.

#### Exclusion criteria

- If the carious tooth shows signs or symptoms of irreversible pulp pathology or loss of vitality including:
  - The presence of a sinus
  - Tenderness to percussion
  - Buccal tenderness
  - Pathological mobility
  - Severe sensitivity
  - Evidence of pathology on a periapical radiograph

### Outcome measures

#### Primary outcome

Clinical

- Sustained tooth vitality

Economic

- Incremental net benefit (WTP minus costs) over a modelled lifetime horizon

#### Secondary outcomes

Clinical

- Pulp exposure during caries removal;
- Progression of caries;
- Tooth restoration failure and re-restoration

Patient-centered

- Dental pain; need for dental pain relief;
- Health status; Oral Health-Related Quality of Life
- Oral health behaviours
- Patient satisfaction

Economic

- General population preferences,
- Willingness to pay
- NHS and patient perspective costs collected over three years follow up, and modelled over a lifetime horizon
- QALYs and incremental cost per QALY over three years follow up, and modelled over a lifetime horizon
- Incremental net benefits over three years follow-up

## Data collection and processing

Participating dental practices will be expected to maintain a file of essential trial documentation which will be provided by the Trial Coordinating Office Dundee (TCOD).

Participants who lose capacity to consent during the study will be withdrawn. Identifiable data already collected with consent would be retained and used in the study. No further data will be collected or any other research procedures carried out on or in relation to the participant.

### Baseline characteristics

At baseline information will be collected on participants’ socio-demographic characteristics, generic health-related quality of life, oral health-related quality of life, oral health behaviours, and time and travel costs through a participant questionnaire. Oral health status and caries experience (DMFT) will be calculated by the clinician. Bitewing radiographs and periapical radiographs (if clinically needed) will be collected as part of routine care but copies will be provided to the trial team to provide a measure of the extent of caries and confirm exclusion of signs of pulp pathology. A case report form (CRF) will record the details of treatment including caries removal, restoration placed, and resources required to deliver the respective treatments. Any modification of or deviation from the intervention will be recorded on the CRF to inform the clinical outcomes.

### Clinical outcomes

Clinical outcomes will be recorded on the CRF completed by the clinician at the end of each course of treatment until the end of the trial. It will record clinical and participant-reported signs and symptoms of tooth pain, the findings of any radiographs taken, the reason for and detail of any dental treatment provided for included teeth. Radiographs taken during the trial will be taken according to good practice guidelines based on caries risk at intervals between 6 and 24 months. Clinicians will take a bitewing radiograph at one year post-treatment, in accordance with Faculty of General Dental Practitioners (FGDP) guidelines [23] since included patients are considered at high caries risk, at the follow-up visit and the trial team will request a copy for treatment adherence monitoring. If a participant has not received any post-intervention visit by 4 months before the end of the follow-up period, they will be contacted by the dental practice and offered an appointment. All radiographs will be assessed by a clinical researcher who is blinded to treatment allocation. Digital radiographs will be forwarded via the secure trial management system and digital images of wet films made.

***Sustained tooth vitality ***will be collected at routine dental visits, recorded in the CRF and used in a time- to-event framework defined as the time from randomisation to root canal treatment or extraction due to loss of vitality, the primary time point of interest is three years. Sustained tooth vitality will be determined by the absence of root canal treatment or extraction due to loss of vitality and the absence of clinical signs and symptoms of pulp death including evidence from radiographs.

***Pulp exposure during caries removal ***will be recorded by the clinician at the time of intervention. If soft tissue within the tooth is exposed this will be detected visually by bleeding and or a pink or red spot.

***Progression of caries ***will be clinically and radiographically assessed by the clinician at each visit as per national guidelines. An independent blinded assessor will also evaluate the radiolucent area on radiographs taken between baseline and follow-up.

***Tooth restoration failure and re-restoration ***will include the reason and subsequent treatment and will be collected at routine visits in a similar fashion to the primary outcome.

### Participant reported outcomes

Patient focused outcomes will be recorded pre-randomisation at the baseline visit and on an annual postal or online follow-up questionnaire until 3-years post-randomisation (except when indicated otherwise).

Dental pain and need for dental pain relief will be recorded on the annual patient questionnaire. Dental pain will be measured using a Numerical Pain Rating Scale (NPRS) [24]. Health status will be assessed using the generic EQ-5D-5L, consisting of five dimensions of Health-Related Quality of Life (HRQoL) [25]. Oral Health Related Quality of Life will be assessed using the Oral Health Impact Profile 14 (OHIP-14). OHIP-14 is the most commonly used validated oral HRQoL measure and has been used successfully in previous HTA trials [26]. Oral Health behaviours will be assessed at baseline and 3-years post-randomisation using questions about type of toothbrush, brushing twice a day for 2 min, frequency of interdental cleaning and behaviour after brushing.

### Outcomes from data linkage with routine administrative NHS health records

Dataset: Management Information and Dental Accounting System (MIDAS) [27] collated information on all child and adult primary care dental practice appointments and treatments in Scotland (including Childsmile practice prevention items)**.**

Trial participant data will be linked to individual health records from MIDAS database, tooth-specific data on specific treatments (restorations, root canal therapy, and extractions).

### Economic outcomes

NHS perspective costs will be collected using a combination of clinician reported data using CRFs (for the costs of intervention delivery and caries treatment provided), data linkage to routinely collected dental claims (including tooth level data where possible) from the respective countries (ISD in Scotland, NHS BSA in England) and participant reported contact with non-dental NHS services for problems related to their teeth. Participant perspective costs will be obtained from NHS claims data (for treatment co-charges) and through self-reported information through participant completed questionnaires in practice and annually. Time, travel and lost productivity costs associated with required dental care will be collected through a questionnaire with a sample of respondents, collected in the first or second annual questionnaire. EQ-5D-5L data from annual questionnaires will be used to calculate QALYs.

General population preferences will be obtained from a discrete choice experiment (DCE with an online representative sample of the UK general population. The DCE will be used to elicit willingness to pay (WTP) for SCR, CCR and patient relevant short and longer term outcomes (e.g. need for further treatment). The DCE will be used to predict intervention uptake.

### Process evaluation

A mixed-method process evaluation will complement the outcome evaluation and examine implementation, mechanisms of impact and context as per MRC guidance [25]:

#### Implementation

The process through which the intervention (SCR or CCR) is delivered in dental practices, what is delivered in different practices and by whom, the fidelity and adaptation of the protocol and the resources used.

#### Mechanisms of impact

How the intervention is received by patients (acceptability) and how clinician/patient interactions trigger change in approaches to caries removal and placement of restorations and any unintended effects.

#### Context

Through examining how external factors including dental contracts and the use of skill mix influence the delivery of the intervention and its outcomes. The process evaluation will include the self-report questionnaires as described above, analysis of trial outcomes data (including CRFs) and qualitative interviews with patients, dental professionals and other stakeholders such as managers of corporate bodies and dental service commissioners.

### Process evaluation: qualitative interviews—SCRiPT participants

A sample of SCRiPT participants will be interviewed to explore their experiences of the intervention. A purposive sample will be used to ensure a range of participants in terms of age, patterns of dental attendance and tooth vitality or not. Potential participants for interview will be identified by the research team and they will be sent a letter of invitation along with a participant information sheet about the interview. Those who are willing to take part in the interview will return an expression of interest form to the research team. If they expressed an interest in being interviewed, they will then be contacted by the researcher to arrange a suitable time and location to hold the interview. Before the start of each interview the researcher will obtain informed consent. Recruitment will continue until no new themes emerge. Previous similar studies have involved 10–15 patients in qualitative interviews [25]. The interviews will be guided by the concept of patient acceptability using a topic guide developed by the research team. The interviews will be audio-recorded and transcribed verbatim by a professional transcription service who will be approved as vendors by the sponsor. The data will be analysed using framework analysis as it provides a pragmatic approach which produces results that can be easily incorporated into mixed-method studies, process evaluations and RCTs [28–30] (see details below).

### Process evaluation: qualitative interviews—dental stakeholders

A sample of dental stakeholders will be interviewed during the set-up phase to explore their views about the usual care they provide and how they envisage delivering the intervention, and during the trial to explore their experiences of delivering the intervention within their own setting. Individual stakeholders may take part in one or two interviews and may be interviewed at both time points to consider experiences of the trial in relation to expectations. Dental stakeholders will be purposively sampled based on role, geographical location, the types of dental contract they are working under, previous experience of differing restorative techniques and, for interviews carried out once sites have opened, their levels of engagement with the trial. Potential stakeholder participants will be selected, with dental professionals selected from the list of all practices involved in SCRiPT. The sample will include those who had recorded instances of having deviated from the clinical protocol for a variety of reasons, as these cases are of particular interest. Recruitment will continue until no new themes emerge. Previous similar studies have involved 25–30 stakeholders in qualitative interviews [31]. Potential participants for interview will be identified by the research team and they will be sent a letter of invitation along with a participant information sheet about the interviews. Before the start of each stakeholder’s first and any subsequent interview the researcher will obtain and record informed consent using appropriate method, e.g. verbal, written, digital.

The interviews will be guided by the theoretical domains framework (TDF) [32] which has been used previously in implementation research to understand the motivations, cognitions and behaviours of dental professionals when implementing evidence-based practice [31–33]. A topic guide based on the TDF has been developed by the research team. The interviews will be audio-recorded and transcribed verbatim by a professional transcription service who will be approved as vendors by the sponsor. Framework analysis based on the TDF will be used.

The analysis will involve the following stages: identifying initial themes, labelling the data, sorting the data by theme and synthesising the data. The analysis will be conducted by an experienced research associate with support from grant-holders. In addition, during the analysis, regular meetings will be held between the research team to discuss the emergent themes and consider the implications of these for the implementation and delivery of the intervention.

### Scheduling of events

Data will be collected as detailed in Table 1.

**Table 1:** Scheduling of events

	Screening	Baseline (initial treatment visit)	At time of intervention/dental visits	Annual Questionnaire	36 months	Other
Assessment for eligibility	o					
Informed consent		o				
Socio-demographic characteristics and eligibility for free treatment		o				
Clinical status (DMFT)		o				
Sustained tooth vitality			o		o	o
Pulp exposure during caries removal			o			
Caries progression dental pain relief			o		o	o
EQ-5D-5L		●		●	●	
OHIP-14		●		●	●	
Patient satisfaction				•	●	
Oral health behaviours		●			●	
NHS perspective primary dental care resource use and cost			o			∇
NHS perspective use of other NHS services (GP, A&E etc.)			o	●	●	
Patient perspective unit costs of time and travel^A^				●		
Patient perspective costs (private care, NHS co-charges etc.)				●	●	∇
General population preferences						⊕
Willingness to pay						⊕

^A^Costs of time and travel will be collected from a randomly selected subset of participants, across the different annual questionnaire time-points. Each selected participant will complete the questions once only

o: Dental Practice-CRF

●: Questionnaire

∇: Data linkage to routine administrative datasets, ongoing over trial duration, at the end of the study, and for longer term follow-up

⊕ DCE, administered once online to a nationally representative sample of the UK general population

## Study within a trial

Poor retention of participants recruited to clinical trials is a known problem which can reduce statistical power, bias estimates of intervention effects and reduce the credibility of trial results [34]. SCRiPT will investigate whether giving participants a welcome letter on entry to the trial improves questionnaire response rates and retention across the trial’s follow-up. The letter will welcome participants to the trial, reiterate the participant journey (summarising information provided in the patient information leaflet), identify the location of the participant’s study tooth and list the expectations for participation i.e. the need to return the trial questionnaires. Allocation to receive or not receive the letter will be randomised following recruitment into the trial by the trial office and will be 1:1. The intervention group will be issued with a letter within 2 months of consent to participate. The control group will not be issued with a letter.

## Analysis plan

### Sample size calculation

The sample size calculation is event-based. We aim to detect an absolute improvement in sustained tooth vitality at three years of 12% from 80% (CCR) to 92% (SCR). The control rate of 80% is based upon the existing evidence base, clinical experience and confirmed by Scottish tooth-level routine data. As described in Sect. 2.1 above, the current evidence base gives rates of pulpal exposure that are higher than those seen in UK NHS and therefore our rate of sustained tooth vitality will be higher than observed in those studies. Bjørndal 2017 [12] presented a tooth vitality rate of approximately 80% at 3 years for two-step selective restoration in lesions that were deeper than those in this trial. The two-step procedure is more similar to a CCR than the SCR in our trial. The Scottish tooth specific data demonstrated that of 459,648 teeth that required a three surface restoration in 2013, 6.6% had received further endodontic treatment or were extracted at 3 years follow-up. These teeth however, could be at any level of dentine involvement and it is not possible to identify the level of involvement in the routine data. Therefore, as the study is interested in only teeth with involvement in the pulpal third of dentine, it has been estimated that the number of events may be three times larger. There is little empirical evidence to inform the expected size of an important effect. A target difference of 12% was judged clinically important and plausible by both dentists and PPI partners. The target difference of 12% was also informed by the difference observed in Bjørndal 2017 [12] which was 13.7% at 5 years. However, as this is a patient RCT there are concerns around contamination (participants receive the intervention that they were not randomly allocated to). The team conservatively have allowed for up to 25% contamination and therefore the sample size for SCRiPT aims to detect an absolute improvement of 9%; from 83 to 92% at three years. Recruitment of 623 participants (71 events) will detect a hazard ratio of 0.42 between experimental and control strategies and provide, using the log-rank test, assuming an exponential failure rate, 90% power at a 2-sided 5% significance level. The calculation also assumes 17 months of recruitment, a minimum of 33 months of follow-up and a 30% follow-up attrition at the end of year three. Recruitment is assumed to be staggered and builds in seasonal variation. We used the Stata package ARTSURV for calculations [35].

### Proposed analyses

Demographic and baseline characteristics will be summarised and displayed in tables for all randomised patient participants using appropriate summary statistics. All analyses will initially be performed on an intention -to-treat basis, although use causal methods for the analysis of the primary outcome to estimate efficacy in the presence of cross- over between treatment arms will also be used. Analysis will be fully specified in a Statistical Analysis Plan.

### Primary outcome

The primary endpoint, sustained tooth vitality, will be analysed within a time-to-event framework using a Cox proportional hazards model, adjusted for minimisation covariates and a random effect for clinician included. The treatment effect will be summarised by the hazard ratio with a 95% confidence interval. The estimated survivor function will be graphed.

### Secondary outcomes

Secondary clinical time-to-event outcomes (progression of caries; tooth restoration failure and re-restoration) will be analysed in a similar manner to the primary outcome. Pulp exposure will be analysed using logistic regression adjusted for minimisation covariates and a random effect for clinician included. Secondary outcomes reported by participants will be analysed using mixed effects generalised linear models (using the appropriate link function for the outcome distribution) for repeated measures. Models will make use of data available at all time points, adjusted for minimisation covariates, fixed effects for treatment and (nominal) time points, and random effects for participant and clinician. The primary time point of interest will be three years post-randomisation. Treatment effects will be estimated using a treatment-by-time interaction and presented with 95% confidence intervals.

### Planned subgroup analysis

Subgroup analyses on the primary outcome will explore the possible modification of treatment effect by age and number of lesions. This will be done by including treatment-by-factor interactions in the model and they will be classified as exploratory analyses.

### Proposed frequency of analysis

From the internal pilot phase we will report estimates of recruitment rates and potentially eligible participants, together with appropriate confidence intervals. There are no planned interim analyses of the primary or secondary outcomes; one final analysis will occur at the end of follow-up.

### Missing data

Strategies with proven effectiveness in improving retention will be used to minimise missing data [36]. For participants who do not return or are censored early in the trial, dental practices will be contacted to find out potential reasons for missing data if they are available.

Participants with no event will be censored at their last visit with sustained tooth vitality. It is anticipated that a small proportion of participants will not return at all, and a further proportion will be censored early in the trial because they do not come back to their clinician. The team will consider how robust the findings are to these missing data using multiple imputation approaches under an assumption of missing at random, and using pattern mixture models if appropriate. In participants with more than one eligible tooth, we will repeat our primary analysis with a clustering effect for participant.

Routinely collected data will be considered to supplement missing outcome data if deemed appropriate. We will undertake a validation analysis comparing (primary outcome) CRF data with routinely collected tooth-level data which will also provide the opportunity for secondary and economic outcome data analysis if the data are valid. This will be detailed in a data linkage analysis plan.

### Transfer of data

Data transfer will adhere to the processes detailed in the CHaRT Standard Operating Procedure book.

The data will be de-identified before it is securely transferred. The data will be transferred using ZendTo. This is a secure web-based service which can be used to securely transfer data between colleagues both inside and outside the university. Files are stored on and accessed from the University’s secure server, and all files are checked for viruses when they are uploaded. The de-identified data will be encrypted before it is sent with ZendTo, using Office365 AES-256 encryption.

However, sometimes it may be necessary to transfer files on CD or USB stick. In such cases, a robust system logging the receipt of sent items must be in place either for a CD coming into the centre or leaving the centre—for example, by registered mail or courier, requiring signature on delivery. As with electronic data, the data on the CD/USB stick should be encrypted and password protected using an acceptable standard of encryption currently available (at least 256-bit encryption).

Data transferred from CHaRT to external parties will be subject to approval by the Project Management Group using a data request form.

Access to data linked to NHS Scotland routine administrative data for outcome analysis will be undertaken within the NHS Scotland National Safe Haven infrastructure [37].

## Economic evaluation

A full economic evaluation will be conducted. This will include a trial-based analysis at three years follow-up and a decision model to assess economic value over an extrapolated lifetime horizon. The primary economic outcome will be net benefit, WTP minus cost (NHS and patient perspective) evaluated in a cost–benefit analysis (CBA) framework, modelled over a lifetime horizon. CBA is chosen as the preferred framework because of concerns that generic QALY measures are not sufficiently sensitive to capture the value of important outcomes in dental care. Cost Utility Analysis (CUA) reporting cost per QALY will also be conducted as a secondary economic analysis to comply with NICE guidelines for technology appraisal.

### Estimation of costs

Routinely collected dental claims data will be used to assess the costs to the NHS, and co-charges to patients of NHS provided dental treatments. Routine datasets will be supplemented with data collected using CRFs in the dental practice at each visit. Remaining resource use data, NHS and patient perspective costs directly related to dental problems will be collected using patient questionnaires. NHS costs of providing SCR and CCR will be based on the appropriate NHS contract-based payments to clinicians in the respective UK regions for the base case analysis. Contract payments detail the cost burden to the NHS but may not capture the full opportunity cost of time spent and materials used to deliver the interventions. Therefore, a micro-costing approach will also be used as a secondary analysis. Resource use data for the intervention micro-costing will include staff resource use and time, and consumables with information collected in a detailed CRF at the point of intervention delivery. Patient perspective costs will include patient co-charges for NHS treatments, time and travel costs, time-off work, privately purchased care and self-purchased dental care products. The participant time and travel cost questionnaire will be sent to a random sample (N = 347) of trial participants. The required sample reflects the minimum number of respondents contributing complete data that is required to enable a multi-variable regression to estimate costs according to a range of predictor characteristics of the sample. The minimum sample size is calculated from the formula N ≥ 50 + 8(k) [38] where k is the number of independent categories of the model assuming linear additive effects and no interactions. For k = 15 explanatory variables in the model, the minimum number of fully complete responses to the questionnaire is N ≥ 50 + 8(15) ≥ 170. Assuming a questionnaire non-response rate of 30% and a further item non-response rate that would preclude running the model of 30% of returned questionnaires (as per the IQuaD study), the minimum number of questionnaires that need to be sent to obtain the required sample is N = 347 (170/0.7/0.7). The questionnaire will be administered to a random sample of respondents split across the one and two-year annual follow-up time-points. The information collected will also be used to validate an external prediction model of time and travel costs that can be used in future research. Incremental NHS and patient perspective costs for SCR vs CCR will be estimated using generalised linear regression models with appropriate specification of distributions for cost and outcomes data and adjustment for baseline covariates.

### Estimation of benefits

Willingness to pay (WTP) for the cost–benefit analysis (CBA) will be elicited from a discrete choice experiment (DCE). The DCE will be conducted with a nationally representative sample of the UK general population, using online panel surveys. The DCE will explicitly value preferences for provision of SCR and CCR, together with a range of plausible outcomes from the trial (e.g. sustained tooth vitality, longer term risk of repeat treatments and tooth loss). The DCE will examine the trade-offs between the potential benefits and risks of different treatment strategies. A cost attribute will be included to enable calculation of WTP. The DCE will also be used to assess the acceptability of SCR and CCR and predict uptake according to patient characteristics (investigating issues of preference heterogeneity for treatment). The target sample size for the DCE (N = 1067) is calculated using Dillman, 2007 [39], using an estimate of the population of interest (i.e. the UK general adult population) =  ~ 52 million), a conservative estimate of variation in the answers for the question of interest of 0.5, and an assumed margin of error of 3% in line with public opinion research, with a confidence level of 95%. A further sample of 100 respondents will be sought for a pilot study of the DCE. As a secondary objective, we will measure benefits in terms of QALYs gained, based on patient responses to the generic EQ-5D-5L health related quality of life measure.

### Trial based economic evaluation

Economic evaluations typically take the form of cost-utility (i.e. cost per QALY). However, in the context of dentistry, there are concerns that generic EQ-5D based QALYs lack the sensitivity to capture the processes and outcomes of care that are of value to patients and decision makers. Different perspectives of benefits will therefore be evaluated (Willingness to pay (WTP) for the interventions and outcomes, WTP for dental health outcomes only, and Quality Adjusted Life Years (QALYs). Costs will be evaluated from an NHS dental, all NHS, and participant perspective. Costs and benefits will be combined to estimate incremental net benefit (WTP minus costs), incremental net dental health benefit, and incremental cost per QALY gained for SCR vs, CCR over the three year trial follow up period. Deterministic sensitivity analyses will be undertaken to test the impact of assumptions and analysis methods on results. Subgroup analysis will be conducted at the region level if data allow in order to explore the potential impact of different payment systems across the UK regions on results. Results will be plotted on the cost–benefit and cost-effectiveness planes to illustrate the impact of sampling uncertainty on results.

### Decision modelling

The trial results will be extrapolated over a lifetime horizon using a de novo tooth-level Markov model. The model will be built as a cohort model, but we will retain the option to move to a more flexible micro-simulation model if appropriate. Results will be reported using the same net benefit framework as the within trial analysis. The final model structure and health state definition (e.g. loss of tooth vitality, tooth-restoration-complex failure, caries progression, and tooth loss) will be developed in conjunction with dental and patient experts. Survival analysis methodology will be used to assess the time to transition between health states, with survival curves fitted over an extended time frame (patient’s life time) to extrapolate the time to event data from the trial. The survival analysis will be supplemented with data from cohort studies and literature reviews to complete population of model transition probabilities and relative effects as required. Cost data (NHS and patient perspective) for health states will be sourced from the trial data and routine data sources (PHS / BSA). Benefits in terms of WTP will be sourced directly from the DCE for specific health states (e.g. WTP to avoid loss of tooth vitality, tooth-restoration-complex failure, caries progression or tooth loss), together with WTP tariffs from previously conducted DCE studies [40]. Sensitivity analyses will explore the impact of key assumptions on results. Gaps in the evidence base will be identified and their potential impact on efficiency (net benefit) explored through sensitivity analysis. Results will be reported according to the same cost and benefit perspectives as detailed for the trial-based analysis. Findings will be illustrated using cost-effectiveness and cost–benefit acceptability curves. A value of information analysis will be undertaken to determine the need for future research to resolve any residual decision uncertainty and an expected value of partial perfect information (EVPPI) analysis will be used to prioritise future cost-effectiveness research objectives.

## Ethical conduct of the trial

The trial will run under the auspices of the trial office in Dundee Dental School and CHaRT in the University of Aberdeen. CHaRT is a fully registered Clinical Trials Unit with extensive expertise in running multicentre RCTs. Both institutions are committed to the highest standards of research governance and conform to all relevant governance guidelines and codes of practice as detailed in the Research Governance Framework and ICH guidelines for Good Clinical Practice (GCP). Favourable ethical opinion for the SCRiPT study was confirmed by the North of Scotland Research Ethics Committee (REC) on the 6th January 2020. The trial will be conducted according to the principles of GCP provided by Research Governance Guidelines. Annual progress reports, end of Trial declaration, and a final report will be submitted to the Sponsor and the North of Scotland REC within the timelines defined in the regulations.

The CI will ensure, through the TSC and Sponsor that, adequate systems are in place for monitoring the quality of the trial and appropriate expedited and routine reports, to a level appropriate to the risk assessment of the trial.

A study information leaflet will be given to each potential participant to inform them of the anticipated risks and benefits of taking part in the study. In particular, the trade-offs between possible short- term benefits and long-term risks will be explained. Informed consent will be obtained from the participants in all practices, including the parent or guardian of 12–15 year-old participants, by an individual who is trained in GCP. Patients will be given sufficient time to accept or decline involvement and are free to withdraw from the study at any time.

## Data protection and archiving

Patients will be reassured that all data which are collected during the course of the research will be kept strictly confidential. All personal data will be pseudonymised and processed in accordance with the General Data Protection Regulation Act 2018. The relevant research documentation will be archived at the University of Dundee for at least five years after completion of the trial as required by the applicable regulatory requirement(s).

## Governance arrangements

Research Governance applies to everyone working in the Dental Health Services & Research Unit and CHaRT. As such, all research will be conducted within the appropriate legislative and regulatory environment and in accordance with GCP. All staff involved in the trial at the two centres will have undertaken appropriate GCP training (to a level of knowledge that reflects their exposure to the principles). The three main groupings that contribute to the governance arrangements for this study are: the Trial Management Committee; an independent Trial Steering Committee (TSC); and an independent Data Monitoring Committee (DMC). The Trial Steering Committee (TSC) includes an independent Chairperson (Professor Paul Colthard, Queen Mary University) and other independent members (P Duncan, R Ladwa, I Soulsby, C Vernazza, H Worthington and P Burns) and will oversee the trial. The TSC also comprises a selection of the co- applicants including the Principal Investigators (Clarkson and Ramsay), the trial statistician and the Director of CHaRT. There will only be two voting members drawn from any of the co-applicants. The TSC will meet annually throughout the course of the study.

The Data Monitoring and Ethics Committee (DMEC) will be chaired by A Maguire and include B Chadwick and R Playle. It will meet early in the trial to agree terms of reference and other procedures and will likely have further meetings at 9, 24 and 36 months. The DMEC will report any recommendations to the Chair of the Steering Committee.

The University of Dundee has agreed to act as sponsor. As such, the TCOD will undertake to communicate promptly and effectively with the sponsor to satisfy and reassure the sponsor that the sponsor’s obligations on the authorisations, the financing and the progress reporting (including emerging safety data) of the trial are being met. This may include providing comprehensive information before the start of a trial for the purposes of risk assessment for the sponsor.

All data will be managed in accordance with GDPR regulations. Information Governance approval for data linkage to NHS Scotland routine administrative datasets will be obtained from NHS Scotland Public Benefit and Privacy Panel for Health and Social Care.

### Arrangements for day-to-day management of the trial

The TCOD based in the Dundee Dental School at the University of Dundee will provide day to day support for the clinical centres and sites. The trial office through the trial manager and other administrative positions will provide a hub for dissemination of administrative and clinical support activities for the trial. The trial manager, trial administrator and trial tecretary at the TCOD will take responsibility for the day to day collecting, collating, handling and entering data for the participant completed postal questionnaires, including organising all aspects of the questionnaires (mailing, tracking, and entering returned data using the study web-based data entry portal).

CHaRT, Health Services Research Unit, Aberdeen University will provide the database applications and IT programming for the TCOD, and host the randomisation system, co-ordinate the patient follow-up questionnaires, provide experienced trial management guidance, and take responsibility for all statistical aspects of the trial (including interim reports to the TSC and DMEC). The PIs (GDPs) will be responsible for recruiting participants (including initiating the randomisation) and performing all clinical outcome assessment.. An Operations Management Group (OMG) led by the Trials Manager, will meet weekly in the early stages at the TCOD to ensure smooth running of the trial, troubleshooting issues as they arise, and ensuring consistency of action across the participating centres. CHaRT staff in Aberdeen will join this group as required, by teleconference.. The study will be supervised by a Project Management Group (PMG). The co-chairs of this group will be the co-chief investigators and will consist of grant holders, representatives from the TCOD and CHaRT. The PMG will meet at least monthly however meetings may be more frequent. In addition, the PMG will also meet at the annual Trial Steering Committee meeting. A Trial Management Committee will meet annually and be chaired by the Principal Investigators and include co-investigators and key members of the TCOD and CHaRT. Their remit will be to oversee the progress of the trial, and they will report to the independent TSC.

As a result of the COVID-19 pandemic the trial committees have moved from face-to-face meetings to teleconferencing and will continue to do so in line with governmental guidelines.

### Safety concerns

SCRiPT involves procedures and treatment which are well established in current NHS clinical practice and use. In this trial, the following events are anticipated and are captured as primary or secondary outcomes rather than being captured through adverse event or serious adverse event reporting processes.

- failure of tooth vitality with associated signs or symptoms (e.g. pain, infection, swelling, periodontitis)
- further treatment required

As this reflects the routine care the trial is designed to measure.

In addition, all deaths (any cause) are also recorded by the trial office. Events that are serious but are not related to caries treatment in the trial tooth will not be recorded as SAEs.

### Sponsorship

The University of Dundee is the sponsor of the research. The sponsor has had no role in the design of the study and will have no role in the collection, analysis, and interpretation of the data and in writing of any future manuscript.

Contact Details:

Patricia Burns.

Tayside Medical Science Centre, Ninewells Hospital & Medical School.

Dundee, DD1 9SY, UNITED KINGDOM.

Telephone: 01382 383297.

E-mail: TASCgovernance@dundee.ac.uk.

### Finance

The study is supported by a grant from the National Institute of Health Research (NIHR) Health Technology Assessment Program (17/127/07).

## Publication

The results of the study will be reported first to study collaborators. A main report will be drafted by the project management group and circulated to all clinical coordinators for comment before a final version is considered for publication by the steering committee.

### Dissemination

Any major proposed changes to the protocol will be communicated with sponsor, the North of Scotland Research Ethics Committee and the HTA.

The findings of the trial will be disseminated widely through professional, primary care, public and scientific routes. The results of the trial will be communicated directly to all participating dental practices who will be invited to attend the SCRiPT conference to showcase the results and work done by the practitioners involved. The results of the trial will be used to update Cochrane reviews and clinical guidelines as published by NICE, SIGN and Scottish Dental Clinical Effectiveness Programme and the online training resource will be made available to learning institutions across the UK. In addition, it is hoped to produce a range of actionable knowledge tools to encourage implementation of the trial results. The cost effectiveness elements of SCRiPT and patient related outcomes will be of high importance to the NHS policy and decision makers, including the UK’s four Chief Dental Officers and as such this trial has the potential to impact decision making for the general dental community both nationally and internationally. To enable this research to be embedded as an output and impact on future decision making we will draw on the extensive networks of the research team who are well connected, respected and cover a vast number of professional fields and demonstrate our ability to actively participate in creating a Global Evidence Ecosystem for Oral Health as aspired to by the MAGIC Project [41]. Many of the research team members are part of the academic teaching community for both national and international undergraduate and postgraduate programmes. Through formal and informal channels and established teaching community networks, we will actively encourage Dental Schools to embed the research findings and clinical implications into teaching.

## Milestones for the SCRiPT trial

Dental practice recruitment began at month 3. Patient recruitment has been delayed due to the effects of COVID-19 and has therefore not commenced on time. We predict that patient recruitment will take place over an 18-month period. Follow up assessments will take place three years after recruitment and delivery of intervention.

## Discussion

The SCRiPT Trial is an NIHR HTA funded trial being undertaken across the UK and will begin to address the lack of high-quality evidence to aid dental practitioners, patients and policy makers in their decision making. As a pragmatic, multi-centre, randomised, open trial with blinded outcome evaluation, SCRiPT aims to eradicate the uncertainty that exists among dental practitioners when treating deep carious lesions by testing the interventions in the environment that they will most often be delivered in, dental primary care.

In order to ensure the results of this trial are widely applicable the geographical areas that are included in the SCRiPT Trial have been selected to yield a cross-section of practices, operating in a range of different environments and circumstances (e.g. high, middle or low income communities, rural and urban, method of remuneration for GDPs (capitation and fee for item of service or a banded payment system based on Units of Dental Activity (UDA)).

The study team is multidisciplinary and broad-based, and will be led by the teams at the Dental Health Services Research Unit, Dundee and the Centre for Healthcare Randomised Trials in Aberdeen. This will ensure that whilst the trial design and conduct is of the highest standard, it remains practical and pragmatic at all times.

## Supplementary Information

**Additional file 1.** Patient information leaflet.

**Additional file 2.** Consent forms.

## Data Availability

The final trial data datasets will be available from the Chief Investigator Jan Clarkson on reasonable request.

## Abbreviations

AE: Adverse Event
CCR: Complete Caries Removal
CHaRT: Centre for Healthcare Randomised Trials
CI: Chief Investigator
CONSORT: Consolidated Standards of Reporting Trials
CRF: Case Report Form
CTU: Clinical Trial Unit
DCE: Discrete Choice Experiment
DMC: Data Monitoring Committee
DMS: Data management system
EQ-5D-5L: EuroQOL five dimensions questionnaire
GCP: Good Clinical Practice
GDP: General Dental Practitioner
HRQoL: Health Related Quality of Life
HSRU: Health Services Research Unit
HTA: Health Technology Assessment
ICF: Informed Consent Form
ISD: Information Statistics Division
ISF: Investigator Site File
ISRCTN: International Standard Randomised Controlled Trial Number
IVR: Interactive Voice Response (randomisation
OMG: Operations Management Group
MRC: Medical Research Council
NCT: National Clinical Trial
NHS: National Health Service
NIHR: National Institute Health Research
PI: Principal Investigator
PIL: Patient Information Leaflet
PMG: Project Management Group
PPI: Patient and Public Involvement
PQ: Patient Questionnaire
QALY: Quality Adjusted Life Year
RCT: Randomised Controlled Trial
R&D: Research and Development
REC: Research Ethics Committee
SAE: Serious Adverse Event
SAP: Statistical Analysis Plan
SCR: Selective Caries Removal
SD: Standard Deviation
SOP: Standard Operating Procedures
TCOD: Trial Coordinating Office Dundee
TMF: Trial Master File
TSC: Trial Steering Committee
UK: United Kingdom
UKCRC: United Kingdom Clinical Research Collaboration
UoA: University of Aberdeen
