# Supplementary material for: Selective Caries Removal in Permanent Teeth (SCRiPT) for the treatment of deep carious lesions: a randomised controlled clinical trial in primary care
Source: BMC Oral Health. 2021 Jul 9;21:336. doi: 10.1186/s12903-021-01637-6 (PMC8267238; doi:10.1186/s12903-021-01637-6)
Supplement: Supplementary file 1 — Additional file 1. Patient information leaflet. [file 12903_2021_1637_MOESM1_ESM.docx]

**
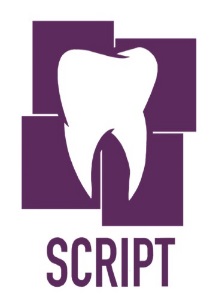
**

NIHR HTA SCRIPT STUDY

Participant Information Leaflet (Parts A, B and C)

**SCRIPT** - **Selective Caries Removal In Permanent Teeth**

IRAS number 268742

**INVITATION TO TAKE PART**

We would like to invite you to take part in a research study. The SCRIPT study is looking at the best way to treat a tooth with deep decay. It is comparing two treatments for deep decay in permanent teeth. Although both treatments work well, it is it is not clear which of these treatments are better for keeping your tooth healthy. For participants, the study will run for 3 years (study treatment data will be obtained for a longer period). The study team aims to recruit around 623 participants from dental practices across the UK.

Before you decide if you would like to take part it is important for you to understand why the research is being done and what it will involve.

Please take time to read the following information carefully. Feel free to talk to you dentist or other people about taking part in the study if you wish. Ask your dentist if there is anything that is not clear or if you would like more information

**Part A: Patient Journey**

**Are you interested in taking part in a dental research study?**

**You could take part at your treatment appointment!**

**If you choose to take part**

You will complete and sign a

**Consent Form**

You will fill in a **Questionnaire** about you and your teeth

You will either have

**Treatment 1** or **Treatment 2**

(You won’t know which one)

**Treatment 1:**

Complete removal of decay and a filling

**Treatment 2:**

Selective removal of decay and a filling

**You will have regular check-ups as usual with your dentist.**

At your check-up 1 year after your filling, you will have an X-ray done to check your filling.

At each check-up, your dentist will take a note for the research study to identify progress being made.

If you have not had a check-up within 3 years after treatment, you will need to have one.

You will be asked to complete a **Questionnaire** for the research study

1 year, 2 years, and 3 years

after your filling was done

**Interested in knowing more?**

Please read Part B and Part C of this leaflet and talk to the Dental Practice Team

To thank you for your support, you will receive a £15 gift voucher at the beginning and at the end of the study.

**If you choose not to take part**

You will have your treatment

(usually Treatment 1)


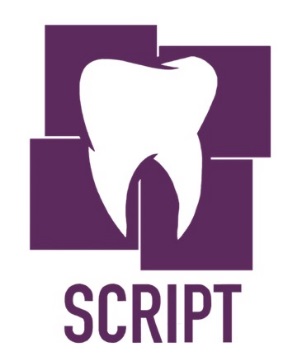


We would like to invite you to take part in an important dental study called SCRIPT.

**What is the SCRIPT study?**

The SCRIPT study is trying to find out the best way to treat a tooth with deep decay (the decay is called **caries** by dentists). It is looking at two ways to treat the caries or deep decay.

Treatment 1: remove all the decay and use a filling to fill the hole in the tooth.

Treatment 2: remove only some of the decay near the top of the hole in the tooth and use a filling to fill the hole in the tooth.

The dentists in the SCRIPT study are taking part because, although both treatments work well, it is not clear which of these treatments are better for keeping your tooth healthy.

**Why are you asking me?**

Your dentist believes either of these two treatments would be suitable for you.

We are asking a number of people around the country who also have deep decay (caries) to take part in the SCRIPT study. By collecting information, the study will help us find the answer to the question – Which treatment is better?

**What treatment will I get?**

You will get **only one** of these two treatments:

1. All the decay in your tooth is taken out and then a filling put into the hole.
2. Only some of the decay in your tooth is taken out and then a filling put into the hole.

Half of the people taking part in the SCRIPT study will get Treatment 1. The other half will get Treatment 2. If you take part, you can’t choose which treatment you get – and neither can your dentist – it is decided by a computer.

We use a computer to help us make the types of people in the 2 groups similar. Then we can know that any differences we measure between the groups are to do with the type of treatment, rather than anything else.

**What is the difference between the two treatments?**

Both treatments work well. However, currently it is not clear which one works better overall. This is why we need to do this study.

Treatment 1: This makes sure the deep decay is removed. Your dentist will work very carefully and use an X-ray to guide them. However, because the decay is so deep, there is a chance that the tooth’s nerve could be damaged when removing the decay. If the tooth’s nerve does become damaged, there is then a 7 in 10 chance that the tooth will have to have root canal treatment or will need to be removed within the next 5 years.

Treatment 2: This means that some of the decay is removed and a filling is then put in to stop the decay from getting worse. There is a reduced chance that the nerve will be damaged when only some of the decay is taken out. If only some of the decay is taken out it is possible that the tooth will still need more treatment later.

This study will help us work out which filling is better overall.

**What’s in it for me?**

You will receive treatment for your tooth decay. You might not gain any additional benefit from taking part. However, you will be directly helping us to inform the treatment of future patients with deep tooth decay. The results of the study will help plan effective services offered by the NHS in the future.

**What will happen if I say yes?**

1. **Your dentist will ask you to sign a consent form.**

This form is used to record that you understand the study and what will happen. You will be given a copy of the form to keep, as well as this information leaflet.

1. **You will be given a short questionnaire about yourself and your teeth**.

Your dentist will enter some of this information about you into the study computer and it will tell them whether you will get treatment 1 or 2. Your dentist will not tell you which treatment you get.

1. **You will then have your treatment and go home**.
2. **You will come back for check-ups and treatment by your dentist as normal**. This will include having an X-ray of your teeth to check your filling after a year.
3. **In 1 year, 2 years and 3 years’ time, we will send you a questionnaire (by email or post).** The questionnaire will ask you some more questions about your teeth and yourself.
4. On the consent form you will also be asked if you would consider taking part in other related research. This includes an interview study where you would be asked to talk about your experience of dental treatment in the SCRIPT study.

**Who will know I am in this study?**

You will be given a special identification number and any information we collect for the study will be stored using this number, rather than using any personal details. This means that only the people who are treating you, or who need to contact you, will know who you really are. In this research study we will use information from you and your dental records. We will only use information that we need for the research study. We will let very few people know your name or contact details, and only if they really need it for this study.

Everyone involved in this study will keep your data safe and secure. We will also follow all privacy rules.

At the end of the study we will save some of the data for future research. We will make sure no-one can work out who you are from the reports we write. The information pack tells you more about this.

**Do I have to say yes?**

No you don’t. If you decide to say no, nobody will mind, and you and your dentist can talk about what treatment you will have.

**What happens if I change my mind?**

You can change your mind about taking part any time you want, and the study will stop contacting you. You will still be treated by your dentist as they normally would. If you do change your mind about taking part, please tell your dentist or the SCRIPT study team.

**Does COVID-19 impact on this research?**

COVID-19 doesn’t change either of the treatments in SCRIPT themselves. However, dentists are taking extra precautions to prevent the spread of COVID-19 with all their treatments. All safety precautions will be followed with regards to COVID-19 during this research.

**Who is running the SCRIPT study?**

This study is being run by a team of experienced dentists and researchers, led by Professor Jan Clarkson at the Dundee Dental School. The study is sponsored by the University of Dundee and has been funded by the National Institute for Health Research.

**How can I find out more?**

If you have any questions or worries about the information in this leaflet or anything else related to the SCRIPT study please speak to your dentist. Further details of the study are available online at

<https://w3.abdn.ac.uk/hsru/script>

**
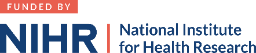
**SCRIPT Study Office, Level 9, School of Dentistry, University of Dundee, Dundee Dental Hospital, Park Place, DUNDEE Scotland DD1 4HN Tel: 01382 383817, Email: script@dundee.ac.uk

**Who is eligible to take part?**

Adults and young people aged 12 years and over, who receive some or all of their care through the NHS and have been diagnosed with deep decay in a permanent molar that requires treatment may be eligible. Eligibility will be confirmed by your dentist. We want to compare two treatments for this kind of dental decay to see which is better in terms of keeping your tooth healthy.

**Who has reviewed this study?**

All research in the NHS is looked at by an independent group of people, called a Research Ethics Committee (REC), to protect your interests.  This study has been reviewed and given favourable opinion by the North of Scotland Research Ethics Service.

**How have patients and the public been involved in this study?**

Members of the public are represented on the research team throughout the study.  They have helped with drafting this patient information leaflet.

**What will happen to the results of this study?**

The results of the study will be used to make recommendations on treatments for patients with deep decay.  We shall publish the results of this study in scientific journals and present the information at appropriate meetings.  You will not be identified in any publication of results of the study. We will let you know the results of the study when it is finished unless you tell us that you do not wish to know.

**What happens when the research study stops?**

Your dentist will continue to provide your dental care after the study has ended. Your future dental care will be at the discretion of your own dentist. You will be able to access the results of the study once they are published and we will provide you with details about how to access this information.

If for any reason the study ends earlier than expected, we will let you know and your continuing care will carry on as normal.

**What if relevant new information becomes available?**

Sometimes during the course of a research project, new information becomes available about the treatment that is being studied.  If this happens, the SCRIPT Study Office staff will contact you to let you know about the choices available to you.  However, we are not aware that any new, relevant information is likely to become available before the end of this study.

**What will happen if I don't want to carry on with the study?**

You can decide at any time to withdraw from the study. This decision will not affect the standard of care you are receiving now or in the future. If you make this decision, you should continue attending appointments with your dentist as part of your standard care.

If you decide to withdraw from this research study, we will keep and continue to use all your previously collected data. We will, however not collect any further data about you. This information will remain confidential and will not be used for any other purpose.  To safeguard your rights, we will use the minimum personally-identifiable information possible.

**Will my taking part in the study be kept confidential?**

Yes.  We will follow ethical and legal practice and all information which is collected about you for the purpose of this research study will be handled in strict confidence and securely stored by the Universities of Aberdeen, Dundee and Sheffield.  Only specified members of the research team will have access to your information.

**Personal information and routine national data**

University of Dundee will collect information about you for this study from you and your dentist. We will need to use information from you and from your dental records for this research project.

This information will include

- your name
- NHS number/ CHI number/ Health and Care Number
- contact details (address, email address, telephone numbers)
- date of birth.

We will only use this information to conduct this study. We will use this information to do the research or to check your records to make sure that the research is being done properly.

People who do not need to know who you are will not be able to see your name or contact details. Your data will have a code number instead.

We will keep all information about you safe and secure.

We will review national health registers to find out about relevant treatments you have received and the progress of your tooth both during and after the study has ended. To carry this out we need to send some information about you to national health registers (e.g. NHS Business Services Authority, HSC Business Services Organisation (BSO), NHS National Services Scotland (NSS), Public Health Scotland, Office of National Statistics (ONS), NHS Digital). This information will then be matched to their records about dental treatments you have received, and then returned to the University of Aberdeen where it will be anonymised. All information would be sent and stored securely throughout the process.

Once we have finished the study, we will keep some of the data so we can check the results. We will write our reports in a way that no-one can work out that you took part in the study.

**What are your choices about how your information is used?**

- You can stop being part of the study at any time, without giving a reason, but we will keep information about you that we already have.
- If you choose to stop taking part in the study, we would like to continue collecting information about your health from central NHS records and your dentist. If you do not want this to happen, tell us and we will stop.
- We need to manage your records in specific ways for the research to be reliable. This means that we won’t be able to let you see or change the data we hold about you.
- If you agree to take part in this study, you will have the option to take part in future research using your data saved from this study.

**Where can you find out more about how your information is used?**

You can find out more about how we use your information

- at [www.hra.nhs.uk/information-about-patients/](https://www.hra.nhs.uk/information-about-patients/)
- our leaflet available from the research sponsor <https://www.dundee.ac.uk/information-governance/dataprotection/>
- by asking one of the research team at your dentist or the SCRiPT Study Office
- by sending an email to [script@dundee.ac.uk](mailto:script@dundee.ac.uk) or
- by ringing us on 01382 383817

**What if there is a problem?**

If you are concerned about your participation in the study you have the right to discuss your concern with a researcher involved in carrying out the study or a dentist involved in your care. If you have a complaint about your participation in the study first of all you should talk to a researcher involved in the study or your dentist. You can also make a formal complaint to the Complaints Officer for your local NHS Health Board or NHS Clinical Commissioning Group.

The University of Dundee holds and maintains policies of insurance for legal liabilities arising from the study

If you are harmed due to someone’s negligence, then as a patient of the NHS, you may have grounds for legal action. You may have to pay for your legal costs yourself.

**The following information explains how health researchers use information from patients. If you are asked to take part in research, you can ask what will happen in the study.**

**What is patient data?**

When you go to your GP or hospital, the doctors and others looking after you will record information about your health. This will include your health problems, and the tests and treatment you have had. They might want to know about family history, if you smoke or what work you do. All this information that is recorded about you is called patient data or patient information.

When information about your health care joins together with information that can show who you are (like your name or NHS number) it is called identifiable patient information. It’s important to all of us that this identifiable patient information is kept confidential to the patient and the people who need to know relevant bits of that information to look after the patient. There are special rules to keep confidential patient information safe and secure.

**What sort of patient data does health and care research use?**

There are lots of different types of health and care research.

If you take part in a clinical trial, researchers will be testing a medicine or other treatment. Or you may take part in a research study where you have some health tests or answer some questions. When you have agreed to take part in the study, the research team may look at your medical history and ask you questions to see if you are suitable for the study. During the study you may have blood tests or other health checks, and you may complete questionnaires. The research team will record this data in special forms and combine it with the information from everyone else in the study. This recorded information is research data.

In other types of research, you won’t need to do anything different, but the research team will be looking at some of your health records. This sort of research may use some data from your GP, hospital or central NHS records. Some research will combine these records with information from other places, like schools or social care. The information that the researcher collects from the health records is research data.

**Why does health and care research use information from patients?**

In clinical trials, the researchers are collecting data that will tell them whether one treatment is better or worse than other. The information they collect will show how safe a treatment is, or whether it is making a difference to your health. Different people can respond differently to a treatment. By collecting information from lots of people, researchers can use statistics to work out what effect a treatment is having.

Other types of research will collect data from lots of health records to look for patterns. It might be looking to see if any problems happen more in patients taking a medicine. Or to see if people who have screening tests are more likely to stay healthier.

Some research will use blood tests or samples along with information about the patient’s health. Researchers may be looking at changes in cells or chemicals due to a disease.

All research should only use the patient data that it really needs to do the research. You can ask what parts of your health records will be looked at.

**How does research use patient data?**

If you take part in some types of research, like clinical trials, some of the research team will need to know your name and contact details so they can contact you about your research appointments, or to send you questionnaires. Researchers must always make sure that as few people as possible can see this sort of information that can show who you are.

In lots of research, most of the research team will not need to know your name. In these cases, someone will remove your name from the research data and replace it with a code number. This is called coded data, or the technical term is pseudonymised data. For example, your blood test might be labelled with your code number instead of your name. It can be matched up with the rest of the data relating to you by the code number.

In other research, only the doctor copying the data from your health records will know your name. They will replace your name with a code number. They will also make sure that any other information that could show who you are is removed. For example, instead of using your date of birth they will give the research team your age. When there is no information that could show who you are, this is called anonymous data.

**Where will my data go?**

Sometimes your own doctor or care team will be involved in doing a research study. Often, they will be part of a bigger research team. This may involve other hospitals, or universities or companies developing new treatments. Sometimes parts of the research team will be in other countries. You can ask about where your data will go. You can also check whether the data they get will include information that could show who you are. Research teams in other countries must stick to the rules that the UK uses.

All the computers storing patient data must meet special security arrangements.

If you want to find out more about how companies develop and sell new medicines, the Association of the British Pharmaceutical Industry has information on its [website](http://www.abpi.org.uk/).

**What are my choices about my patient data?**

- You can stop being part of a research study at any time, without giving a reason, but the research team will keep the research data about you that they already have. You can find out what would happen with your data before you agree to take part in a study.
- In some studies, once you have finished treatment the research team will continue to collect some information from your doctor or from central NHS records over a few months or years so the research team can track your health. If you do not want this to happen, you can say you want to stop any more information being collected.
- Researchers need to manage your records in specific ways for the research to be reliable. This means that they won’t be able to let you see or change the data they hold about you. Research could go wrong if data is removed or changed.

**What happens to my research data after the study?**

Researchers must make sure they write the reports about the study in a way that no-one can work out that you took part in the study.

Once they have finished the study, the research team will keep the research data for several years, in case they need to check it. You can ask about who will keep it, whether it includes your name, and how long they will keep it.

Usually your hospital or GP where you are taking part in the study will keep a copy of the research data along with your name. The organisation running the research will usually only keep a coded copy of your research data, without your name included. This is kept so the results can be checked.

If you agree to take part in a research study, you may get the choice to give your research data from this study for future research. Sometimes this future research may use research data that has had your name and NHS number removed. Or it may use research data that could show who you are. You will be told what options there are. You will get details if your research data will be joined up with other information about you or your health, such as from your GP or social services.

Once your details like your name or NHS number have been removed, other researchers won’t be able to contact you to ask you about future research.

Any information that could show who you are will be held safely with strict limits on who can access it.

You may also have the choice for the hospital or researchers to keep your contact details and some of your health information, so they can invite you to take part in future clinical trials or other studies. Your data will not be used to sell you anything. It will not be given to other organisations or companies except for research.

**Will the use of my data meet GDPR rules?**

GDPR stands for the General Data Protection Regulation. In the UK we follow the GDPR rules and have a law called the Data Protection Act. All research using patient data must follow UK laws and rules.

Universities, NHS organisations and companies may use patient data to do research to make health and care better.

When companies do research to develop new treatments, they need to be able to prove that they need to use patient data for the research, and that they need to do the research to develop new treatments. In legal terms this means that they have a ‘legitimate interest’ in using patient data.

Universities and the NHS are funded from taxes and they are expected to do research as part of their job. They still need to be able to prove that they need to use patient data for the research. In legal terms this means that they use patient data as part of ‘a task in the public interest’.

If they could do the research without using patient data they would not be allowed to get your data.

Researchers must show that their research takes account of the views of patients and ordinary members of the public. They must also show how they protect the privacy of the people who take part. An NHS research ethics committee checks this before the research starts.

**What if I don't want my patient data used for research?**

You will have a choice about taking part in a clinical trial testing a treatment. If you choose not to take part, that is fine.

In most cases you will also have a choice about your patient data being used for other types of research. There are two cases where this might not happen:

1. When the research is using anonymous information. Because it’s anonymous, the research team don’t know whose data it is and can’t ask you.
2. When it would not be possible for the research team to ask everyone. This would usually be because of the number of people who would have to be contacted. Sometimes it will be because the research could be biased if some people chose not to agree. In this case a special NHS group will check that the reasons are valid. You can opt-out of your data being used for this sort of research. You can ask your GP about opting-out, or you can [find out more](https://www.hra.nhs.uk/information-about-patients/).

**Who can I contact if I have a complaint?**

If you want to complain about how researchers have handled your information, you should contact the research team. If you are not happy after that, you can contact the Data Protection Officer. The research team can give you details of the right Data Protection Officer.

If you are not happy with their response or believe they are processing your data in a way that is not right or lawful, you can complain to the Information Commissioner’s Office (ICO) ([www.ico.org.uk](https://ico.org.uk/)  or 0303 123 1113).

**Thank you for taking the time to read this information leaflet. We hope that it has been helpful in enabling you to decide if you would like to participate in the SCRIPT Study. Please ask us if you have questions or would like more information about the study.**
