# Supplementary material for: Selective Caries Removal in Permanent Teeth (SCRiPT) for the treatment of deep carious lesions: a randomised controlled clinical trial in primary care
Source: BMC Oral Health. 2021 Jul 9;21:336. doi: 10.1186/s12903-021-01637-6 (PMC8267238; doi:10.1186/s12903-021-01637-6)
Supplement: Supplementary file 2 — Additional file 2. Consent forms. [file 12903_2021_1637_MOESM2_ESM.docx]

**SCRIPT STUDY** - **Selective Caries Removal in Permanent Teeth**

PARTICIPANT ***CONSENT*** FORM

IRAS ID 268742

Please

**INITIAL**

each

point

**Investigators**: Prof Jan Clarkson, University of Dundee; Prof Craig Ramsay, University of Aberdeen

**Sponsors**: University of Dundee

If you **agree** to the following sentences, please ***initial*** next to each statement:

1. I have read the Participant Information Leaflet (parts A, B, C) v4 08.02.2021 for the SCRIPT Study. I have had the opportunity to think about the information, ask questions and my questions have been answered.
2. I understand that it is my choice to take part. I also understand that I can change my mind at any time in the future and without giving a reason and understand doing so will not affect my dental care or legal rights.
3. I understand that personal information that identifies me that is collected during the Study, together with my personal contact details, will be confidentially and securely stored by the Universities of Dundee, Aberdeen and Sheffield. I agree that the research team can use my contact details to send me study questionnaires and to contact me by phone, post, email or text regarding the Study.
4. I agree that personal information (such as my name, date of birth, home postcode and NHS/CHI number) can be used to find out other information about my dental treatment that is collected by the NHS, including information from national dental payment databases (e.g. NHS Business Services Authority, NHS National Services Scotland (NSS), Public Health Scotland, Office of National Statistics (ONS), NHS Digital).
5. I agree that relevant sections of my dental notes and dental information collected during the study may be looked at by people directly involved in the study (at the Universities of Dundee and Aberdeen or in the NHS) where it is relevant to my taking part in this research.
6. **I agree to take part in the SCRIPT study.**

**OPTIONAL (You do not have to give consent to these points to take part in SCRIPT)**

1. I am willing to be contacted in the future about participating in other ethically approved research.
2. I am willing to be contacted in the future for long-term follow-up.
3. I agree that information collected about me may be shared anonymously with other researchers to support future research.

**PARTICIPANT SIGNATURE**

Your Signature Name in BLOCK CAPITALS Date

**For Dental Practice Staff Use Only**

SIGNATURE OF PERSON TAKING CONSENT

Job Title Name in BLOCK CAPITALS Signature Date

SCRIPT Study Office Dundee, Level 9, School of Dentistry, University of Dundee, Dundee Dental Hospital, Park Place, DUNDEE Scotland DD1 4HN

Tel: 01382 383817, Email: [script@dundee.ac.uk](mailto:script@dundee.ac.uk)

**COPIES: Original (top copy)**: For Study Office. **Yellow copy:** For practice site file. **Pink copy:** For patient participant.

**SCRIPT STUDY** - **Selective Caries Removal In Permanent Teeth**

PARTICIPANT (under 16) **CONSENT** FORM

IRAS ID 268742

Please

**INITIAL**

each

point

**Investigators**: Prof Jan Clarkson, University of Dundee; Prof Craig Ramsay, University of Aberdeen

**Sponsors**: University of Dundee

If you **agree** to the following sentences, please ***initial*** next to each statement:

1. I have read the Participant Information Leaflet (parts A, B, C) v4 08.02.2021 for the SCRIPT Study. I have had the opportunity to think about the information, ask questions and my questions have been answered.
2. I understand that it is my choice to take part. I also understand that I can change my mind at any time in the future and without giving a reason and understand doing so will not affect my dental care or legal rights.
3. I understand that personal information that identifies me that is collected during the Study, together with my personal contact details, will be confidentially and securely stored by the Universities of Dundee, Aberdeen and Sheffield. I agree that the research team can use my contact details to send me study questionnaires and to contact me by phone, post, email or text regarding the Study.
4. I agree that personal information (such as my name, date of birth, home postcode and NHS/CHI number) can be used to find out other information about my dental treatment that is collected by the NHS, including information from national dental payment databases (e.g. NHS Business Services Authority, NHS National Services Scotland (NSS), Public Health Scotland, Office of National Statistics (ONS), NHS Digital).
5. I agree that relevant sections of my dental notes and dental information collected during the study may be looked at by people directly involved in the study (at the Universities of Dundee and Aberdeen or in the NHS) where it is relevant to my taking part in this research.
6. **I agree to take part in the SCRIPT study.**

**OPTIONAL (You do not have to give consent to these points to take part in SCRIPT)**

1. I am willing to be contacted in the future about participating in other ethically approved research.
2. I am willing to be contacted in the future for long-term follow-up.
3. I agree that information collected about me may be shared anonymously with other researchers to support future research.

**PARTICIPANT SIGNATURE**

Your Signature Name in BLOCK CAPITALS Date

**For Dental Practice Staff Use Only**

1. I confirm that this participant consent has been received from this participant’s parent or legal guardian.
2. SIGNATURE OF PERSON TAKING CONSENT

Job Title Name in BLOCK CAPITALS Signature Date

SCRIPT Study Office Dundee, Level 9, School of Dentistry, University of Dundee, Dundee Dental Hospital, Park Place, DUNDEE Scotland DD1 4HN

Tel: 01382 383817, Email: [script@dundee.ac.uk](mailto:script@dundee.ac.uk)

**COPIES: Original (top copy)**: For Study Office. **Yellow copy:** For practice site file. **Pink copy:** For patient participant.

**SCRIPT STUDY** - **Selective Caries Removal In Permanent Teeth**

PARENT/LEGAL GUARDIAN CONSENT FORM

IRAS ID 268742

Please

**INITIAL**

each

point

**Investigators**: Prof Jan Clarkson, University of Dundee; Prof Craig Ramsay, University of Aberdeen

**Sponsors**: University of Dundee

If you **agree** to the following sentences, please ***initial*** next to each statement:

1. I have read the Participant Information Leaflet (parts A, B, C) v4 08.02.2021 for the SCRIPT Study. I have had the opportunity to think about the information, ask questions and my questions have been answered.
2. I understand that my child has a choice to take part or not. I also understand that my child can change their mind at any time in the future and without giving a reason and understand doing so will not affect their dental care or legal rights.
3. I understand that personal information that identifies my child that is collected during the Study, together with their personal contact details, will be confidentially and securely stored by the Universities of Dundee, Aberdeen and Sheffield. I agree that the research team can use my or my child’s contact details to send study questionnaires and to contact by phone, post, email or text regarding the Study.
4. I agree that personal information (such as my child’s name, date of birth, home postcode and NHS/CHI number) can be used to find out other information about their dental treatment that is collected by the NHS, including information from national dental payment databases (e.g. NHS Business Services Authority, NHS National Services Scotland (NSS), Public Health Scotland, Office of National Statistics (ONS), NHS Digital).
5. I agree that relevant sections of my child’s dental notes and dental information collected during the study may be looked at by people directly involved in the study (at the Universities of Dundee and Aberdeen or in the NHS) where it is relevant to my child taking part in this research.
6. **I agree to my child taking part in the SCRIPT study.**

**OPTIONAL (You do not have to give consent to these points to take part in SCRIPT)**

1. I agree my child can be contacted in the future about participating in other ethically approved research.
2. I agree my child can be contacted in the future for long-term follow-up.
3. I agree that information collected about my child may be shared anonymously with other researchers to support future research.

**PARENT/LEGAL GUARDIAN SIGNATURE**

Your Signature Name in BLOCK CAPITALS Date

**For Dental Practice Staff Use Only**

SIGNATURE OF PERSON TAKING CONSENT

Job Title Name in BLOCK CAPITALS Signature Date

SCRIPT Study Office Dundee, Level 9, School of Dentistry, University of Dundee, Dundee Dental Hospital, Park Place, DUNDEE Scotland DD1 4HN

Tel: 01382 383817, Email: [script@dundee.ac.uk](mailto:script@dundee.ac.uk)

**COPIES: Original (top copy)**: For Study Office. **Yellow copy:** For practice site file. **Pink copy:** For parent/legal guardian
